# The Impact of Insecticide Pre-Exposure on Longevity, Feeding Succession, and Egg Batch Size of Wild *Anopheles gambiae* s.l.

**DOI:** 10.1155/2020/8017187

**Published:** 2020-09-28

**Authors:** Grace Msangi, Moses I. Olotu, Aneth M. Mahande, Anitha Philbert, Eliningaya J. Kweka

**Affiliations:** ^1^Department of Biological Sciences, Mkwawa University College of Education, P.O. Box 2513, Iringa, Tanzania; ^2^Division of Livestock and Human Health Disease Vector Control, Tropical Pesticides Research Institute, Mabogini Field Station, Moshi, Tanzania; ^3^Department of Zoology and Wildlife Conservation, University of Dar Es Salaam, Dar Es Salaam, Tanzania; ^4^Tropical Pesticides Research Institute, Division of Livestock and Human Diseases Vector Control, Mosquito Section, P.O. Box 3024, Arusha, Tanzania; ^5^Department of Medical Parasitology and Entomology, Catholic University of Health and Allied Sciences, P.O. Box 1464, Mwanza, Tanzania

## Abstract

**Background:**

Insecticide resistance among the vector population is the main threat to existing control tools available. The current vector control management options rely on applications of recommended public health insecticides, mainly pyrethroids through long-lasting insecticidal nets (LLINs) and indoor residual spraying (IRS). Regular monitoring of insecticide resistance does not provide information on important factors that affect parasite transmission. Such factors include vector longevity, vector competence, feeding success, and fecundity. This study investigated the impacts of insecticide resistance on longevity, feeding behaviour, and egg batch size of *Anopheles gambiae* s.l.

**Method:**

The larval sampling was conducted in rice fields using a standard dipper (350 ml) and reared to adults in field insectary. A WHO susceptibility test was conducted using standard treated permethrin (0.75%) and deltamethrin (0.05%) papers. The susceptible Kisumu strain was used for reference. Feeding succession and egg batch size were monitored for all survivors and control.

**Results:**

The results revealed that mortality rates declined by 52.5 and 59.5% for permethrin and deltamethrin, respectively. The mortality rate for the Kisumu susceptible strain was 100%. The survival rates of wild *An. gambiae* s.l. was between 24 and 27 days. However, the Kisumu susceptible strain blood meal feeding was significantly higher than resistant colony (*t* = 2.789, df = 21, *P*=0.011). Additionally, the susceptible *An. gambiae* s.s. laid more eggs than the resistant *An.gambiae* s.l. colony (Χ^2^ = 1366, df = 1, *P* ≤ 0.05).

**Conclusion:**

It can, therefore, be concluded that the wild *An. gambiae* s.l. had increased longevity, blood feeding, and small egg batch size compared to Kisumu susceptible colonies.

## 1. Introduction

Malaria is still one of the most prevalent human vector-borne disease that threatens the world's population living in areas where there is a risk of infectious bites with 228 million morbidity and 405 000 mortality cases reported in 2018 [1]. About 90% of all malaria deaths in the world occur in sub-Saharan Africa (SSA) due to a combination of factors such as availability of predominant malaria vectors and parasites, as well as local conducive weather conditions [2–7]. Children under the age of five and pregnant women are the most vulnerable groups [1]. Globally, there are 490 species of *Anopheles*, of which only 70 are considered as potential malaria vectors [8]. Currently, species such as *Anopheles gambiae* s.s*., An. arabiensis, An. merus, An. melus, An. coluzzii*, and *An. funestus* members are the main malaria vectors that are responsible for spread of the disease in SSA [9–15]. So far, vector control measures remain a main intervention strategy for the global malaria eradication programme [16–19]. The core vector control measures that are widely used include long-lasting insecticidal nets (LLINs) and indoor residual spraying (IRS) [1]. The strategy aimed to reduce the risk of malaria infection by targeting indoor biting mosquitoes [1, 20]. Despite the effectiveness of these two interventions in malaria control, they are not sufficient to control residual malaria [21–23]. However, the main challenges associated with LLINs and IRS are insecticide resistance, improper use of the interventions, host behaviour, such as staying outdoor during early night or sleeping outdoor without using protective measures, and vector behaviour including outdoor biting and outdoor resting [21].

Insecticide resistance has now been reported in malaria vector against the four classes of public health insecticides used in malaria vector control, and it was estimated that, in 2016, resistance would have been reported in 71 malaria endemic countries [24] and may continue to threaten sustainability of malaria interventions. So far, pyrethroids have been the only class used for LLINs and contributed for a large proportion of the insecticide used for IRS. Insecticide resistance occurs due to the use of the same chemical insecticides of public health repeatedly [25, 26] or multiple exposures of vectors to different sources of insecticides. It has also been associated with higher doses application than the ones recommended for malaria control [25, 27]. Different resistance mechanisms recorded so far are metabolic resistance, target-site mechanism, and behavioural resistance, as well as cuticle-reduced penetration resistance [25, 27, 28]. In the target-site mechanism, the configuration that occurs in the amino acid leads to a less active or inactive binding site [27–29]. Similarly, malaria vectors also have the ability to produce high amount of enzyme naturally, which enable them to metabolize the insecticide and become functionless [30]. Other insects modify their cuticle or the digestive track by slowing down or prevent the absorption of the insecticides penetrations [31], while behavioural resistance occurs after a long time exposure of insects to an insecticide [24, 27, 32, 33]. The resistance mechanism has been confirmed to help mosquitoes in enduring endless insecticide stress, and when these mechanism actions are high, physiological characteristics such as mosquito longevity, larva development, reproduction, or its blood feeding ability may be affected [34–36]. For example, permethrin has been reported to reduce the blood feeding ability of *An. arabiensis* [37], *An. stephensi*, and *Ae. aegypti* [36]. It significantly reduced the egg batch size up to 100% to the resistant colony of *Ae. aegypti* [38]. The resistance status with multiple blood meals increases the survival of the vectors and tolerance of the resistance with aging [39], while in a normal scenario without multiple feeding, the resistance decreases with aging [32]. Occurrence of insecticide resistance among malaria vectors is an impediment to the disease control efforts. In response to this problem, the World Health Organization has put in place the information systems for insecticide resistance monitoring [40]. Nevertheless, most other important factors that determine the ability of the vector to transmit the parasites are underexplored during the day-to-day insecticide resistance monitoring activities. Such factors include vector longevity, biting behavior, and vector competence of the insecticide-resistant malaria vectors, and their impact on malaria epidemiology is underexplored [41, 42]. To be successfully transmitted, *Plasmodium* parasites must complete their life cycle from gametocytes in the blood meal to sporozoites in the saliva [41]. This means that vectors should live long enough to allow such development of the parasite. Previous studies reported that insecticide resistance genes in vector mosquitoes can cause pleiotropic effects to other genes and, hence, may affect the resistant vectors either negatively or positively by interfering with their fecundity, longevity, and or vector competence [42, 43]. In addition, the insecticide susceptibility test demonstrated mortality beyond the 24 hours of the WHO susceptibility test in resistant mosquitoes, implying the shortening of the parasite transmission potentials [44]. However, studies to explore the interplay between vector resistance with longevity and fecundity are scanty. Most studies have been conducted in a laboratory setting using laboratory-reared colonies. It was important to explore these factors from the natural environment. The present study investigated the impact of insecticide pre-exposure on longevity, feeding behaviour, and egg batch size of *An. gambiae s.l.* from northern Tanzania.

## 2. Materials and Methods

### 2.1. Study Site

This study was conducted at Mabogini Village in Moshi District, Kilimanjaro Region, northeastern Tanzania. The experimental site was located between latitude 03° 21′ S and longitude 37° 20′ E 750 m above the sea level, covering an area of 1600 ha [45]. The site is well known for anthropogenic activities, mainly rice irrigation that contributes to significant breeding sites of mosquitoes [46]. The annual temperature ranges from 18.0°C to 30.7°C, while the average annual rainfall is 525 mm between March and May with a shorter period of rainfall occurring between November and January [47].

### 2.2. Larval Sampling

Mosquito larvae and pupae were collected from rice paddy fields by using a standard dipper (350 ml ladle) and reared in plastic containers [48]. All sampled larvae were morphologically identified and pooled into groups according to their species using morphological keys [49]. The sampled larvae were reared at the Mabogini field station, Moshi, close to rice irrigation schemes.

### 2.3. Rearing of Mosquito Larvae

All collected *Anopheles gambiae* s.l were reared in plastic containers of 18 × 18 × 18 cm with fresh water. Larvae were reared under insectary conditions, temperature 27°C ± 2°C, at relative humidity of 78 ± 2%, and photo phase of 12L: 12D for their growth and development [50]. They were provided with Cerelac and fish food powders on the ratio of 2 : 1 on a daily basis [32]. Pupae were collected using droppers and transferred in emerging cages of 30 × 30 × 30 cm in round metal containers to prevent them from flying away when they emerge into adults. The emerging adult mosquitoes were supplied with cotton wool dipped in 10% glucose solution [48].

### 2.4. Susceptibility Test

Susceptibility bioassays were carried out using insecticide susceptibility kits [51, 52]. Mosquitoes were exposed to papers impregnated with the WHO-recommended discriminating concentrations (*v*/*w*) of (0.05%) deltamethrin and (0.75%) permethrin [52]. Controls were exposed to clean paper impregnated with silicon oil. The female mosquitoes from larvae that were collected from the rice paddy fields 3–5 days after prominence were used for the bioassay test, and the susceptible Kisumu strain was used for reference. The knockdown (KD) rates were recorded at 10, 15 20, 30, 40, 50, and 60 minutes after being exposed to the insecticides and when the KD was less than 80% was observed after 60 minutes [51, 53]. Mosquitoes were, then, transferred into the paper cups and fed with glucose solution. Mortality was recorded after a 24 h holding period; during this time, mosquitoes were provided with a 10% sugar solution [54, 55]. In the experimental rooms, heaters were used to rise temperature when the room temperature dropped, and the floors were wetted to cool the temperature when it rises above the recommended temperature of 25 ± 2°C [52].

### 2.5. Monitoring Longevity of *An. gambiae s.l*

Three-day-old female mosquitoes were exposed to deltamethrin and permethrin (the commonly used pyrethroid insecticide for public health) for an hour; the mosquitoes were, then, placed in a paper cup and were given 10% glucose solution. After 24 hours, the survived mosquitoes were transferred in a cage. They were provided with blood meal. The longevity of a mosquito was monitored until death to mimic the time after exposure of wild population when they contacted insecticide-treated surfaces. Monitoring was after every 24 hr. The dead mosquitoes were counted and removed daily from the cage [56].

### 2.6. Monitoring Feeding Succession of *Anopheles gambiae s.l*

The effect of the insecticide resistance on the feeding succession of *An. gambiae s.l.* was monitored through provision of a blood meal source and monitoring the number successfully fed. The tested vectors were offered blood meal twice a week; the first blood meal was given 48 hours after the susceptibility test was performed. The blood meal was always given in the morning times between 07 : 00 and 09 : 00 h after being sugar-deprived for one hour before the blood meal [57]. Rabbits' fur was shaved dorsal ventrally, and then, they were kept in a restrainer, which limited their movement and exposed the shaven part to mosquitoes to feed for 30 minutes, but the number of successfully fed mosquitoes was recorded after one hour. In order to enhance the feeding process, the cages were covered with dark clothes [58]. Rabbits used to provide the blood meal were obtained from the TPRI laboratory facilities within the Mabogini paddy rice irrigation scheme. The feeding succession was determined by counting the number of mosquitoes that successfully fed on blood during the 30 minutes.

### 2.7. Monitoring Egg Batch Size of *An. gambiae s.l*

After mosquitoes were fed on rabbits for a blood meal, they laid eggs on a wet white filter paper (Whatman No. 1, diameter 9 cm) placed on a plastic transparent Petri dish. Collection of eggs was performed at an interval of three days after every blood meal, and then, the eggs were counted with the aid of a dissecting microscope [59, 60]. After egg counting, the effect of insecticide tolerance on egg batch size of mosquitoes was assessed by comparing the mean number of eggs that were produced from a resistant colony against that of the susceptible colony.

### 2.8. Data Analysis

Data were analyzed using SAS software version 9.3 (SAS Institute Inc. 2008). The generalized linear model (GLM) was used to compare the mean difference between treatments, day and time, and their interactions. The parameters were tested separately for the three treatments (deltamethrin, permethrin, and control) at 95% confidence level. The Bonferroni correction was used to adjust for multimeans comparisons. Fifty percent (50%) and ninety-five percent (95%) knockdown times (KDT_50_ and KDT_95_) for both resistant and susceptible samples were estimated using the log-time probit model. The Kaplan–Meier analysis was used to estimate the longevity of mosquitoes. The comparison between the survivorship curves (susceptible and resistant) was performed using the Wilcoxon signed rank test.

## 3. Results

### 3.1. Insecticides Susceptibility Status

**Figure 1:**
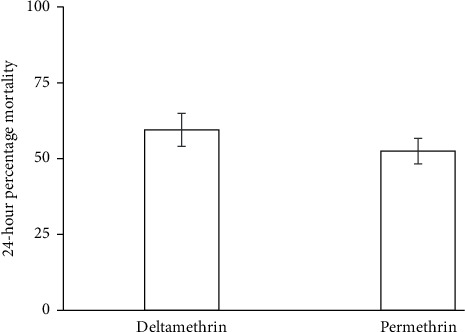
Mortality of wild *Anopheles gambiae* s.l. exposed to deltamethrin and permethrin treatments.

**Figure 2:**
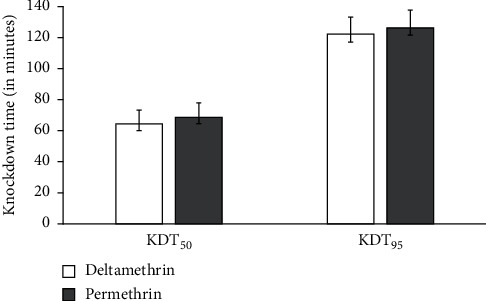
The mean KDT_50_ and KDT_95_ for deltamethrin and permethrin against *Anopheles gambiae* s.l.

**Figure 3:**
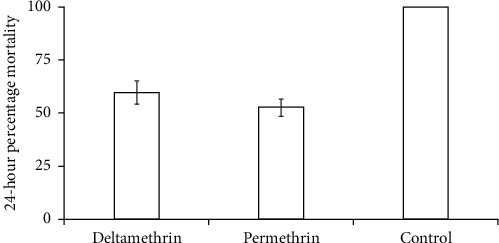
Mortality of wild *Anopheles gambiae* s.l. exposed to different treatments (i.e., deltamethrin, permethrin, and control) (single factor ANOVA, *P*=0.001).

The susceptibility test was conducted using 3–5-day-old unfed female mosquitoes, and in this study, it was found that the resistance status of wild *An. gambiae s.l.* populations was based on decreased mortality rates to permethrin (mortality rate 52.5%) and deltamethrin (mortality rate = 59.5%). The mortality rate for the susceptible reference Kisumu strain was 100%. Also, the study found out that resistance to pyrethroids was matched with increased knockdown times with the KDT_95_ < 20 mins for both pyrethroids. The knockdown time KDT_50_ varied from 64.52 to 68.83 min and KDT_95_ varied from 122.7 to 126. 48 mins for deltamethrin and permethrin, respectively. There was no significant difference, which was observed in the insecticides susceptibility status between deltamethrin and permethrin against *An. gambiae s.l.* (*F*_(1,15)_ = 1.294, *P*=0.599) (Figure 1). However, there was a significant difference in the knockdown time at KDT_95_ as indicated in Figure 2. There was no significant difference in mortality when the wild *An. gambiae s.l.* were treated with deltamethrin and permethrin insecticides, as shown in Figure 1 (*F*_(2,15)_ = 0.923, *P*=0.132), but mortality of the wild *An. gambiae s.l.* differed significantly when the two insecticides were compared with control treatment (susceptible colony) (*F*_(2,15)_ = 4.113, *P*=0.001) (Figure 3).

### 3.2. Longevity of the Insecticide-Resistant Colony against the Susceptible Colony

**Figure 4:**
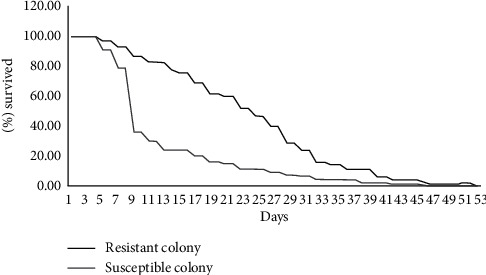
The proportion of resistance (*An. gambiae* s.l.) and susceptible (*An. gambiae* s.s.) survived after blood meal.

Longevity was measured from the time when mosquitoes have been exposed to insecticides and survived and obtain blood meals until the time when they died. Adult female mosquitoes were provided with blood meals twice per week, and 10% glucose were provided in between. The findings revealed that the maximum longevity was 27 days after exposure for the resistant wild *An. gambiae* s.l. and 24 days for the Kisumu susceptible strain. This indicated that the Kisumu susceptible colony lived for a shorter period of time than the wild female *An. gambiae* s.l., and its observed mean longevity was 26 and 20 days in control for the wild *An. gambiae* s.l. and the Kisumu susceptible strain, respectively. The Wilcoxon rank test was used to compare the resistant wild female *An. gambiae* s.l. and susceptible Kisumu strain (*Z* = 6.093, df = 86, *P*=0.001) (Figure 4).

### 3.3. Feeding Succession of the Insecticide-Resistant Colony against the Susceptible Colony

**Figure 5:**
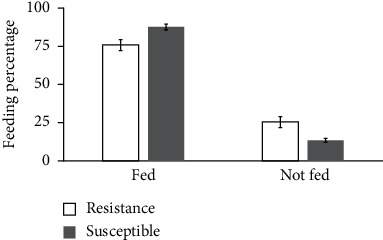
The feeding succession of *Anopheles gambiae* s.s. (Kisumu susceptible strain) and *An. gambiae* s.l. (wild resistant colonies).

Blood feeding ability of wild female *An. gambiae* s.l after being exposed to the insecticides was tested for both strains (i.e., wild *An. gambiae s.l* and Kisumu susceptible strain). They were provided with blood twice per week, and the number of fed and unfed mosquitoes was jotted down. In wild strain, a total of 157 *An. gambiae* s.l that survived the exposure against the two pyrethroids tested were used. It was observed that 119 (75.7%) mosquitoes fed successfully on blood and the remaining 38 (24.3%) did not feed. Furthermore, a total of 400 Kisumu susceptible strain were also fed on blood, and 358 (89.6%) successfully fed on blood, while the remaining 42 (10.4%) did not feed. Statistical analysis indicates that the numbers of female mosquitos fed on blood meals were significantly higher in the Kisumu susceptible strain than in the wild resistant colony (*t* = 2.789, df = 21, *P*=0.011 (Figure 5).

### 3.4. Egg Batch Size of the Insecticide-Resistant Colony versus the Susceptible Colony

**Figure 6:**
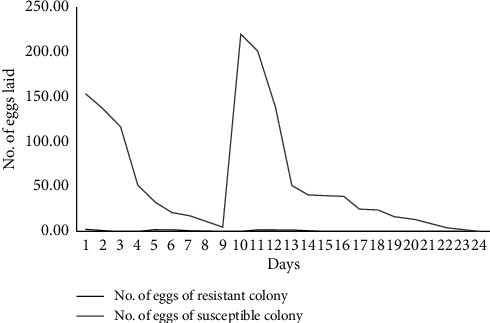
The egg batch sizes of Kisumu susceptible strain (*An. gambiae* s.s.) and (*An. gambiae* s.l.) wild resistant colonies.

The ability of mosquitoes to lay viable eggs and develop into the next generation is another measure of mosquito success in malaria transmission. In this study, the number of eggs that were laid by both strains/colonies was counted and their proportions were compared. The study revealed that the egg batch size of the wild *An. gambiae* s.l. was affected by the two pyrethroids tested. A total of 1409 eggs were laid by both Kisumu susceptible strain and wild *An. gambiae* s.l., whereas 1392 out of these 1409 eggs were laid by the Kisumu susceptible strain and only 17 were laid by the wild *An. gambiae* s.l. that was considered as a resistant colony. Therefore, the study revealed the insecticide resistance impact on the egg-laying ability of the vector (*An. gambiae* s.l.). The Kisumu susceptible colony had laid more eggs than wild female *An. gambiae* s.l., and the difference was significantly different by *X*^2^ = 1366, df = 1, *P* ≤ 0.05 (Figure 6).

## 4. Discussion

The findings of this study show that insecticide resistance has a great impact on susceptibility, egg batch size, longevity, and feeding success of malaria vectors, *An. gambiae* s.l.. The results in this study illustrated that the wild *An. arabiensis* collected from irrigation schemes at Mabogini are resistant to the two pyrethroids (deltamethrin and permethrin). Their tolerance observed to pyrethroids could be elaborated by the fact that the vectors are repeatedly exposed to the pyrethroids in this study area [27, 32, 33, 45, 61, 62]. This is because, in the studied area, the main income-generating activity is agriculture, both animal keeping and crop production (rice), with intensive use of pyrethroid-based insecticides for veterinary, public health, and agricultural purposes, as reported by several studies conducted in the same vicinity [27, 32, 33, 45, 61, 62]. Therefore, mosquitoes might have developed resistance as a result of everyday contact with treated cattle during blood meal because the dominant species in the study area is *An*. *arabiensis*, and this species preferentially feeds on cattle blood as opposed to human blood [63–66]. Furthermore, vector resistance might have resulted as an effect of frequent exposure to insecticides residues in farms from the egg to pupa stages in the rice fields. These results are similar to previous studies that reported resistance in wild *An. gambiae* s.l. to pyrethroids, in lower Moshi, northeastern Tanzania, and in Muleba, northwestern Tanzania [30, 32, 33, 61].

In this study, the longevity of a vector was defined as the time mosquitoes survived the first 24 hours after exposure to deltamethrin/permethrin until actual death. Longevity is an important parameter when considering mosquito competence in transmitting malaria disease; the mosquito must survive long enough so that the *Plasmodium* life cycle will be complete and be transferred to the salivary gland of the vector [57]. The difference in longevity between wild mosquitoes and susceptible colonies showed that the resistant population had shown to survive longer than susceptible ones. These findings were supported with the former studies conducted in a laboratory in Switzerland where increase in longevity was observed in resistant colonies [67]. Similarly, in South Africa, Okoye and others reported longer life span among females of *An. funestus* compared to their susceptible counterparts [65]. However, findings of this study are in contrary to the previous two studies in which longevity was lower on resistant colonies [37]. An increase in the longevity that was observed in the resistant colony in this study could be a threat to the ongoing malaria control efforts and eradication agenda that were set by WHO for the purpose of eliminating malaria globally [67]. Although resistance management strategies often rely on the assumption of reduced fitness in vector populations associated with resistance genes, studies in other insects such as boll weevils, houseflies, and cockroaches do not show differences in fitness between resistant and susceptible strains [68, 69]. Nevertheless, there is no uniform negative effect of fitness costs associated with resistance across species, and the negative performance in one parameter can conceivably be balanced by the positive performance in another [70]. In the wild, various environmental factors including food and temperature affect developmental rates and survivorship of mosquito immature and, subsequently, the adult life span.

The feeding succession is an important parameter of mosquitoes in transmitting disease; therefore, inability to feed on blood can change the transmission dynamics which, in turn, will severely limit their ability to transmit disease [67]. The numbers of fed and unfed mosquitoes were counted after each blood meal for both strains [57]. From the findings of this study, it can be seen that the feeding succession of a wild *An. gambiae* s.l. was reduced and the percentage of unfed *An. gambiae* s.l. was higher in the wild *An. gambiae* s.l. compared to the susceptible colony. This shows that the resistant population of *An. gambiae* s.l. can be denied an opportunity to feed when the host is well protected with LLINs [67]. The results of this study on feeding succession were corroborated by other studies conducted elsewhere [36, 37].

In this study, the resistance character of a vector negatively affected the number of eggs. The wild female *An. gambiae* s.l. laid less number of eggs compared to those which were laid by the susceptible strain. According to Couret and others in their studies, they interpreted a model that illustrated the interaction between larval population size and adult population size and how this model was affected by the number of eggs laid by a mosquito [71]. Smaller number of eggs that would be produced and laid by the resistant colony would have an impact on the number of larvae produced and adult populace that will exist. The number of eggs that were produced by the resistant strain was less compared to that produced by susceptible colony. Similar findings have been reported where resistant colonies showed reduction in egg batch sizes [72], and this information is of public health importance as it translates to reduced vector density.

## 5. Conclusions

The findings of this study have shown the high degree of tolerance to permethrin and deltamethrin insecticides among a wild population of *An. gambiae* s.l. Also, the findings showed an increased longevity of the resistant colony, decreased feeding habit, and decreased egg batch size of the resistant vectors compared to the susceptible colony. These results give an insight on the effects of insecticides resistant on malaria vector species and their impact to the spread of the malaria considering the greater extent nature of pyrethroids resistance in sub-Saharan Africa.

## Data Availability

Comment: Data can be availed upon request from the corresponding author.

## Abbreviations

LLIN: Long-lasting insecticidal nets
IRS: Indoor residual spray
WHO: World Health Organization
TPRI: Tropical Pesticides Research Institute
KDT: Knockdown time
KD: Knockdown.
